# Linking resilience and regulation across system levels in healthcare – a multilevel study

**DOI:** 10.1186/s12913-022-07848-z

**Published:** 2022-04-15

**Authors:** Sina Furnes Øyri, Siri Wiig

**Affiliations:** Faculty of Health Sciences, SHARE - Centre for Resilience in Healthcare, University of Stavanger, Stavanger, Norway

**Keywords:** Resilience, Healthcare regulation, Multilevel, Quality and safety, Risk management

## Abstract

**Background:**

The *Quality Improvement Regulation* was introduced to the Norwegian healthcare system in 2017 as a new national regulatory framework to support local quality and safety efforts in hospitals. A research-based response to this, was to develop a study with the overall research question: How does a new healthcare regulation implemented across three system levels contribute to adaptive capacity in hospital management of quality and safety? Based on development and implementation of the Quality Improvement Regulation, this study aims to synthesize findings across macro, meso, and micro-levels in the Norwegian healthcare system.

**Methods:**

The multilevel embedded case study collected data by documents and interviews. A synthesizing approach to findings across subunits was applied in legal dogmatic and qualitative content analysis. Setting: three governmental macro-level bodies, three meso-level County Governors and three micro-level hospitals. Participants: seven macro-level regulators, 12 meso-level chief county medical officers/inspectors and 20 micro-level hospital managers/quality advisers.

**Results:**

Based on a multilevel investigation*,* three themes were discovered. All system levels considered the *Quality Improvement Regulation* to facilitate adaptive capacity and recognized contextual flexibility as an important regulatory feature. Participants agreed on uncertainty and variation to hamper the ability to plan and anticipate risk. However, findings identified conflicting views amongst inspectors and hospital managers about their collaboration, with different perceptions of the impact of external inspection. The study found no changes in management- or clinical practices, nor substantial change in the external inspection approach due to the new regulatory framework.

**Conclusions:**

The *Quality Improvement Regulation* facilitates adaptive capacity, contradicting the assumption that regulation and resilience are “hopeless opposites”. However, governmental expectations to implementation and external inspection were not fully linked with changes in hospital management. Thus, the study identified a missing link in the current regime. We suggest that macro, meso and micro-levels should be considered collaborative partners in obtaining system-wide adaptive capacity, to ensure efficient risk regulation in quality improvement and patient safety processes. Further studies on regulatory processes could explore how hospital management and implementation are influenced by regulators’, inspectors’, and managers’ professional backgrounds, positions, and daily trade-offs to adapt to changes and maintain high quality care.

## Background

Resilience has become a key priority in healthcare (see definition in Table 1) and persists a theoretical concept that supports the idea of multiple levels’ influence on quality and safety in the complex healthcare system [1–4]. A remaining issue in resilience in healthcare research is to investigate multilevel perspectives, and to include different “theoretical lenses”, as recently encouraged by Wiig & O’Hara [5]. Regulation (see definition in Table 1) and resilience are phenomena often criticized as “hopeless opposites”, but to date, few studies have elaborately investigated the assumption and drawn parallels between the two theoretical lenses [6–10]. Moreover, more knowledge is needed about management responsibilities’ influence on system resilience and managers’ contributions to quality and safety enhancement in healthcare [11]. Despite several previous studies examining organizational adaptive capacity, few studies have linked the resilience potential of adaptive capacity to regulatory activities in a multilevel perspective [4, 6, 7, 12–16]. Research that links resilience across system levels with examination of how regulation affects meso and micro-levels, including how multiple levels influence implementation of changes, is thus requested [5, 17–19]. Improvement initiatives taking the bigger picture of system complexity into account, is lacking, along with challenges related to organizational leadership, and management of quality and safety [20–23]. The application of complex adaptive systems thinking to healthcare systems has demonstrated that outcomes cannot be linearly controlled, and therefore it is prudent to create conditions that enable good outcomes to emerge [24]. Moreover, few attempts in research have been registered to situate micro-level quality improvement within complex system dynamics (where quality is an emergent property) [25]. To summarize the rationale for conducting this study, undertaking the gaps in knowledge: resilience requires adaptive capacity, which requires sufficient autonomy and decision space. Regulation is traditionally seen as opposite to this as it implies prescription [6, 10]. However, there are different approaches to regulation, and there are few studies that have considered how this interface with resilience especially few multilevel studies exploring complex system dynamics [5, 18, 19].

**Table 1:** Healthcare regulation, resilience, and adaptive capacity

**Regulation** was defined in the multilevel case study, as:
1.a general governmental strategy for behavioral modification and control of risk, including external inspection [26–29] 2.one specific Norwegian regulatory framework, “the Quality Improvement Regulation” [30] Different types and strategies of regulation exist, varying with sector and scope. Regulation in this study context is referred to as responsive, performance-based, or process-oriented (see elaboration in Theoretical framework chapter)
**Resilience** was defined in the multilevel case study, as:
• “The capacity to adapt to challenges and changes at different system levels, to maintain high quality care” [4]

Correspondingly, the findings reported in this paper represent a synthesis of perspectives and experiences of the possible links between regulation and adaptive capacity retrieved from three different system levels in the Norwegian healthcare setting. Some key facts about the Norwegian healthcare system [31], including the roles of the different levels, and the interactions between them, are provided in Table 2.

**Table 2:** Key facts about the Norwegian healthcare system

The Norwegian regulatory and supervisory regime [32]
• The Norwegian regulatory and supervisory regime consists of several policymaking and governing bodies, possessing a range of different regulatory strategies • The *Ministry of Health and Care Services* directs the healthcare services and the subordinate bodies by means of comprehensive legislation and annual budgetary allocations • The *Norwegian Directorate of Health* has authority to carry out and implement the Ministry’s health policies and regulations • The *Norwegian Board of Health Supervision* and the *County Governors* are responsible for supervisory activities, and enforcement across the Norwegian healthcare system
The Norwegian specialized healthcare system
• Four regional health authorities have responsibilities to implement national health policies and regulations • The regional health authorities are set to plan, organize, govern, and coordinate all subordinated hospitals in their region (the Health Trusts’ Act) [33]

The multilevel case study reported here, investigated a new regulatory framework called “the Quality Improvement Regulation” designed along a plan, do, study, act (PDSA cycle) systematic [34]. It was developed with the aim of offering support to local, management-based quality and safety efforts in hospitals [30]. The Quality Improvement Regulation replaced the previous regulatory framework, the “Internal Control Regulations” [35]. In Table 3 we illustrate the differences in design of the previous and new regulatory framework and in Table 4 we illustrate the differences in content between the two frameworks. The previous “Internal Control Regulations” and the Quality Improvement Regulation have both a type of performance regulation approach, sharing a nondetailed regulatory design, with similar purpose and scope: requiring any healthcare organization to establish a system for risk management and responsibilities of internal control. However, design and content appear somewhat different, see Table 3 and 4 for explications.

**Table 3:** Contrasts in the *design* of the two regulatory frameworks

The Internal Control Regulations (2002)		The Quality Improvement Regulation (2016)	
**Section**	**Heading**	**Section**	**Heading**
§1	Purpose	§1	Purpose
§2	Scope (organizational)	§2	Scope (organizational)
§3	Internal control	§3	Responsibility for the management system
§4	The content of internal control	§4	Definition
§5	Documentation	§5	Scope and documentation
		§6	Duty to plan (P)
		§7	Duty to implement (D)
		§8	Duty to evaluate (S)
		§9	Duty to correct (A)
		§10	Commencement

**Table 4:** Contrasts in *content* of the previous and new regulatory framework

In contrast to the previous “Internal Control Regulations”, the new Quality Improvement Regulation:
• has a plan, do, study, act structure (the PDSA cycle) [30, 34, 36], • adds management and quality improvement terminology, by explicitly addressing the top hospital management level as judicially responsible for systematic and continuous improvement of quality • specifies delegation of quality improvement work related tasks, by stating that the practical- day to day- implementation is delegated to every management level in the relevant hospital • adds an obligation to annually conduct a systematic evaluation of the organization’s risk management and quality improvement measures
The new Quality Improvement Regulation outlines a set of four main components in hospital risk management and implementation of measures set to improve quality:
• (P) the duty to plan, • (D) the duty to implement, • (S) the duty to evaluate, • (A) the duty to correct
Each major improvement measure or risk reducing measure should:
• operationalize its specific goals, resources, and activities along with the four PDSA components • **consider its measures based on specific contextual conditions: resources, competences, and activities**

The Quality Improvement Regulation was introduced to the Norwegian healthcare system in 2017, after an ordinary process of “hearing” [37]. During the process of hearing, relevant stakeholders in the Norwegian healthcare system were offered the opportunity to comment on the design and content of the new regulation as initially proposed. Stemming from a lack of multilevel research within risk regulation and resilience theoretical approaches, this paper therefore directs its spotlight upon the design, development, introduction, implementation, and management of the Quality Improvement Regulation in the Norwegian hospital setting.

### Aim and research question

This multilevel study draws attention to how risk management and quality improvement efforts in hospitals were facilitated or hampered by governmental influence through regulation and external inspection.

The overall and leading research question was: How does a new healthcare regulation implemented across three system levels contribute to adaptive capacity in hospital management of quality and safety?

By exploring the macro-level in healthcare, the study investigated how efforts to manage and improve quality at the meso and micro-level were impacted by governmental influence and expectations. In turn, the study explored how the macro-level was influenced by meso and micro practices [17]. By exploring the meso-level, the idea was to gain knowledge about how regulatory development and design affected external inspection. Lastly, the rationale for exploring the micro-level was to understand how local level aspects in the investigated hospitals possibly influenced their management of quality, including potential issues with implementation and external inspection. These aspects are evaluated based on a resilience in healthcare perspective.

### Theoretical framework

The multilevel study drew on two main theoretical approaches: 1) risk regulation regimes, including responsive regulation and 2) resilience in healthcare, with emphasis on adaptive capacity.

The conceptual thought behind a risk regulation regime is to explain and analyze different interacting components such as different ideas, rules and practice associated with the regulation of risks [38]. Some parts of the healthcare system are governed by detailed, prescriptive regulations, whilst other regulations aim at securing a certain level of performance. The regulatory principle of “command and control” is for instance the leading principle in many governments’ regulatory risk regimes, with strong emphasis on deterrence and compliance [38]. In these types of regimes, it is assumed that punishment and penalties deter the regulatees from breaking the rules, in contrast to compliance-based approaches associated with strategies such as education, persuasion, and dialogue [38]. Responsive regulation represents a hybrid alternative, with emphasis on contextual flexibility. The essence of responsive regulation is a pyramid of regulatory strategies, with the least coercive strategies at the bottom and the intrusive strategies at the top [39, 40]. One of the responsive strategies is the application of performance-based regulation. The basic principle behind performance-based regulation is to provide an objective with the regulation but leave the operationalization of the content with the regulatees. The strategy of performance-based regulation in a regulatory regime, thus constitutes governmental control *through* enforced self-regulation of risk [10, 41]. This type of regulation mandates and monitors an organization's capacity to self-evaluate, design, and manage its primary processes and internal governance and control systems [42]. Performance-based regulation therefore combines prescriptive regulation with monitoring and management of internal quality and safety processes and performance.

Based on the Quality Improvement Regulation*,* hospital managers at every level are encouraged to choose, plan, and conduct specific measures, and activities to reduce risk and improve quality. Due to the different stakeholders’ views, expectations, and experiences connected to regulatory activity, it was considered relevant to view regulation as a constructive collaboration between governmental and healthcare professional stakeholders [43]. This links with the second theoretical perspective applied in our study, resilience in healthcare, as it may support our efforts to understand how systems can maintain functionality and improve, despite disruptions, surprises, deficiencies, and adverse events [44]. Resilience in healthcare takes a system approach to safety and builds on the assumption that people continually adjust and adapt to variations and shifting circumstances [1–3]. The reported multilevel study rested on the definition developed by the Resilience in Healthcare Research Program (2018–2023) [4] (see Table 1). It moreover implied that we did not analyze the multilevel processes according to the traditional four Hollnagel [45] resilience potentials of monitoring, responding, anticipating, and learning. We considered the “capacity to adapt” perspective most relevant to the analysis of the complexity in uniting regulation and resilience theories in a multilevel context. Moreover, and in a resilience in healthcare perspective, it is not sufficient to single handedly analyze adverse events (Safety I) to establish what is safe or not, it also requires knowledge about how and why processes and activities work well and turn out successfully on a regular basis in healthcare (Safety II) [46]. Research addressing this combined effort to understand safety is thus vital, including examination of complex interaction between various stakeholders [4, 47, 48].

## Methods

### Design

**Fig. 1:**
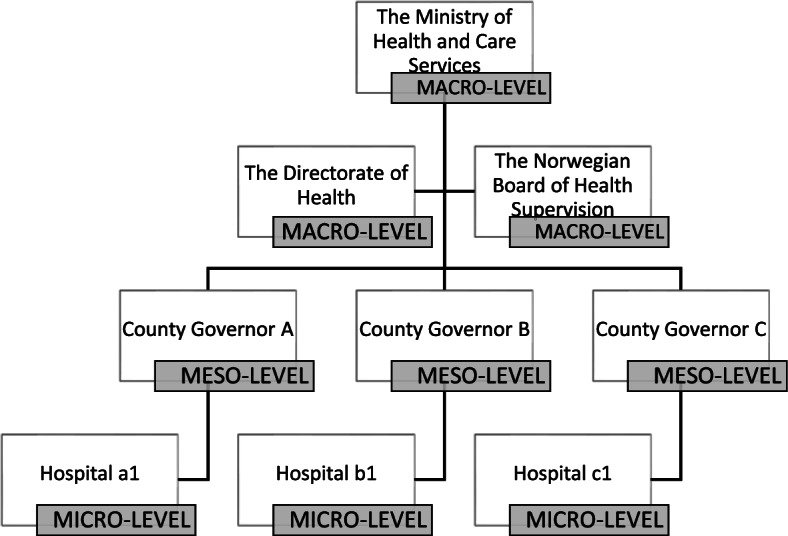
The Norwegian healthcare system explored in this multilevel case study

The study was designed as a multilevel (macro, meso, micro), single embedded case study conducted in the Norwegian healthcare setting during fall 2018 and spring 2019 (see Table 5) [10]. The multilevel study explored the Quality Improvement Regulation’s rationale, expectations, implementation, and management, by involving three levels of stakeholders in the Norwegian healthcare system. The stakeholders included were three governmental regulatory bodies (macro-level), three County Governors with the responsibility of conducting external inspection in the local health trusts (meso-level), and three hospitals (local health trusts) retrieved from two out of four regional health authorities (micro-level) (see Fig. 1). For each level we conducted a sub-study reported in one scientific paper [41, 49, 50].

**Table 5:** Multi-level case study design and data collection

**Level**—unit	**Stakeholder**	**Methods**	**Sub study**
Macro - embedded unit 1	Government officials	Documents approx. 500 pages Interviews: 7 participants	Sub study I [49]
Meso - embedded unit 2	Chief county medical officers; inspectors	Documents approx. 300 pages Interviews: 12 participants	Sub study II [41]
Micro – embedded unit 3	Hospital managers; quality advisors	Interviews: 20 participants	Sub study III [50]

### Data collection

Data was collected by approximately 500 pages of documentary evidence, 29 individual interviews and 3 focus group interviews (10 participants): in total 39 participants (see Table 6 for examples of documentary evidence). Documents were searched and collected by both open Internet searches, as well as by issuing formal letters sent to the Ministry, the Directorate, and the Inspectorate. Governmental laws, guidelines, and White Papers constitute legitimate sources in any design, development, or implementation of governmental regulations. The government documents retrieved from this process hence became key data at the macro and meso level investigation. In terms of exploring the rationale, expectations, implementation and management, other micro level-based internal documents were therefore not in scope.

**Table 6:** Examples of documentary evidence

Publication year	Pages	Title
2002	2	*Internal Control Regulations in the Healthcare Services* [35]
2016	65	*The Prerogative* document for the Quality Improvement Regulation, which stated the narrative of the facts and circumstances of its policies. Formal approval was given in Royal Assent. [51]
2016	3	*The Quality Improvement Regulation.* [30]
2017	57	*Guidelines relating to the Quality Improvement Regulation.* [37]

Participants were mainly selected by purposive sampling and contacted by e-mail. Macro-level participants were seven strategic participants positioned at the Norwegian Ministry of Health and Care Services (the Ministry), the Norwegian Directorate of Health (the Directorate), and the Norwegian Board of Health Supervision (the Inspectorate). Meso-level participants consisted of two chief county medical officers, three assistant chief county medical officers, and seven inspectors, recruited from three County Governors. Micro-level participants consisted of 20 hospital managers or quality advisors selected from different levels at three hospitals, retrieved from two regional health authorities. All interviews were conducted face-to-face at the participant’s workplace, except from three individual telephone interviews [10], and lasted between one hour and one hour and 30 min. All interviews were conducted based on pre-planned semi-structured interview guides, recorded, and transcribed [10]. Field notes were taken during the focus group interviews. Focus group interviews at the meso-level were chosen to explore the discussions and interaction among inspectors. The method was particularly sensible at this system level of the study, as external inspection is carried out by supervisory teams.

### Analysis – synthesis of findings across three sub studies

In the three sub-studies, which resulted in one scientific paper for each system level [10, 41, 49, 50], document data was analyzed by legal dogmatic and qualitative content analysis [52]. Interview data was also analyzed in accordance with principles from qualitative content analysis [52]. For the synthesis of findings across the sub-studies reported in this paper (from the three system levels), we applied a strategy of thematic synthesis [53, 54]. We used the findings in the three sub studies as the basis for synthesizing data across the of macro, meso, micro system levels to “recontextualize” individual findings from each embedded sub-unit in the multilevel case study [10, 41, 49, 50, 53–55].

The interpretive work in the synthesis across the embedded units (macro, meso, micro) focused on possible conflicts in the association between the system of a formal regulatory framework and the way it unfolds into practical contexts of hospital management and external inspection. It is generally demonstrated by cases of governmental requirements designed regardless of complicated reality, often referred to as a gap between *work as imagined* (“blunt end”) and *work as done* (“sharp end”) [19, 28, 46, 56, 57]. Comparing and synthesizing data across three different system levels, resulted in the refinement of three overall themes. This process was done in collaboration among the researchers. The synthesis may contribute to bring the different perspectives together, increase our understanding for complexity challenges to implementation, and thus reduce the gap between *work as imagined* and *work as done* [53, 54]. The overall findings presented in themes from the published papers are summarized by level (macro, meso, micro) in Table 7. Each unit of analysis thus reports findings from either micro, meso, or macro-level. In turn, this paper looks at the evidence altogether through synthesis across these units and system levels [54]. Table 8 displays the three themes as identified in the synthesis.

**Table 7:** Overall findings; presented in themes from the published papers

Macro level
**I Governmental Rationale for Revising the Quality Improvement Regulation**
• Implementation issues with the previous Internal Control Regulations • Lack of management competencies and responsibilities throughout the Norwegian healthcare services • A need to promote quality improvement as a managerial responsibility
**II Expectations of Resilient Capacities**
• Hospitals were expected to *adapt* their risk management to specific context, activities, and conditions • The new regulation might serve as a catalyst for hospital managers to gain a bird’s eye perspective on activities and conditions in their unit; department; clinic • The Government suspected a gap between top-level hospital managers’ priorities and what is done at the clinical level [49]
**Meso level**
**I Changes in Supervisory Work due to the new Quality Improvement Regulation**
• No substantial change in the inspectors’ approach
**II Inspectors’ Work to Apply Regulation and Facilitate Adaptive Capacities**
• Inspectors balanced trade-offs daily, adapting their supervision to specific contexts and cases
**III Learning from Supervision**
• Supervision provides a glimpse into hospital risk management; thus, positive feedback could misleadingly make hospital mangers think that every aspect of their system is fine
**IV Supervisory Impact on Hospital Performance**
• Inspectors demonstrated a general concern about the impact of supervision on hospital performance **V Improvement Potentials in Supervisory Practice** • Inspectors could improve their follow up strategies, use expert inspectors, and add more hospital self-assessment activities, to facilitate learning [41]
**Micro level**
**I Adaptive capacity in hospital management and practice**
• The flexible regulatory design was perceived essential because it is impossible to anticipate every possible event due to different risks and elements of variation and uncertainty
**II Implementation efforts and challenges with quality improvement**
• Hospital managers had too many obligations and lack time to prioritize systematic PDSA methodology • Most physicians worked unconsciously in correspondence with the PDSA methodology
**III Systemic changes**
• Different types of meetings, councils, and committees had been established in recent years • A cultural shift displayed the importance of continuous and structured quality improvement
**IV The potential to learn**
• Difficult to learn from adverse events, as well as from successful outcomes, due to time pressure • Supervision could sometimes be useful; however, inspectors’ recommendations were occasionally difficult or impossible to practically implement [50]

**Table 8:** Themes across sub studies – embedded units 1, 2, 3

Themes across sub studies
**Theme I** A regulatory regime supportive of contextual application does not guarantee actual implementation
**Theme II** Concern about the impact of external inspection on quality and safety in hospital performance
**Theme III** Autonomy and adaptive capacity to tailor quality improvement efforts are imperative for impact

Both documentary evidence and findings retrieved from macro-level participants about regulatory rationale for revising the previous regulatory framework, highlighted expectations towards hospital management and thus formed a foundation for the micro-level investigation. Document analysis at the macro-level contributed to identify the objectives related to expectations directed at the meso-level. In turn, findings about supervisory activity and methods applied in external hospital inspection, informed the data collection that targeted the micro-level. These aspects mentioned were also the backdrop for the construction of the research time wise: by starting “top down”, exploring the macro-level, we gained important insight into the rationale and expectations for the revised regulatory framework. In turn, we had a backdrop that helped us understand the meso-level position “in-between” norm (macro) and practice (micro), as well as it informed our efforts of comparing the normative aspects with the aspects of micro-level managing.

## Results

The synthesis of the findings across these system levels are presented theme wise in the following.

### Theme I—A regulatory regime supportive of contextual application does not guarantee actual implementation

Demonstrated by documentary evidence and macro-level participants, the aim with the Quality Improvement Regulation as revised regulatory framework, was to make it flexible to all types of hospital contexts. This was further confirmed by the meso and micro-levels indicating that managers experienced the revised framework to have a context sensitive design. In turn, the macro-level expected hospital managers to anticipate local risks. Across all levels, the participants highlighted how variation and complexity in healthcare played a major role in why a context sensitive design was important [41, 49, 50]. However, the study found no changes in management practices or clinical practices, despite identification of recent structural and cultural changes to quality improvement in hospitals, such as establishment of different types of patient safety and quality councils, network meetings, and internal audit meetings at the hospitals’ administrative and managerial levels. Nor did we find substantial change in the external inspection approach due to the new regulatory framework, despite a strong recent emphasis on systemic factors in external inspection. Still, inspectors were expected to apply different strategies of the regulatory pyramid [10, 41] in their work, depending on the character and severity of the case.

The Government expected the nondetailed regulatory design to come across as challenging for hospital managers and clinicians, indicating that regulators considered *work as done* when designing the Quality Improvement Regulation. Evidence from the meso-level, backed this up by the chief county medical officers and inspectors agreeing on that the nondetailed regulatory framework provided hospitals with room to maneuver [41]. On the other hand, macro-level as well as micro-level findings demonstrated limited involvement of clinicians in the regulatory design process [49, 50]. Integrated findings also showed a lack of arenas for collaboration, including reflexive spaces to discuss and reflect within and across levels. Lack of micro level involvement during the design process and the implementation process, could hamper quality improvement efforts’ relevance and practical implementation.

Importantly, the integrated findings across macro, meso, and micro levels demonstrated that despite the existence of a regulatory regime that gives the regulatees the option to implement regulations with sensitivity to their own context, a performance-based concept does not guarantee *actual implementation*.

### Theme II—Concern about the impact of external inspection on quality and safety in hospital performance

Meso-level findings demonstrated that external inspection was adapted to specific hospital contexts, with inspectors balancing various regulatory strategies and trade-offs in their evaluations based on the Quality Improvement Regulation [41]. Macro- and micro-level findings however displayed a general concern about the impact of external inspection on quality and safety in hospital performance, related both to the previous regulations and the new regulatory framework [49, 50]. Although some hospital managers described external inspection as helpful in directing attention to certain risks, a lack of trust and motivation to learn from external inspection was reported. This seemed to not have changed along with the introduction of the Quality Improvement Regulation. What meso findings however did demonstrate in terms of supervisory development, was that an increase of case prioritization according to risk potentially could contribute to reduce the pressure of lacking resources at the County Governors offices. Another strategy to reduce the internal manpower resource pressure, was to leave more of the evaluation process with the individual hospitals. Moreover, the County Governors reported this strategy to potentially facilitate a stronger motivation at the hospital level, to learn from adverse events [41]. According to meso and micro participants, inspectors in general could nurture learning by improving their follow up routines and extend their use of expert inspectors with appropriate professional competence in their regulatory enforcement strategies [41, 50].

Meso-level findings moreover indicated that the County Governors were expected to evaluate hospital performance based on certain generic criteria for risk management. The supervisory behaviour expected from the macro-level, did not come as a result from the introduction of the new Quality Improvement Regulation, but was continued as a leading principle from the previous regulations [49]. Thus, the synthesis did not display any fundamental changes in the macro-meso relationship and how the new regulatory framework was expected to be followed up inspection wise, by the County Governors. The hospitals were in turn expected to manage to operationalize these risk management criteria into practical changes. Both meso and micro-level participants however demonstrated organizational variations in these evaluations, arguing that it sometimes related to different descriptions of reality, and thus could lead to external inspection having less impact on hospital performance [41, 50]. The synthesis showed that there still is an unrealized potential to increase learning outcomes from external hospital inspection, regardless of the introduction of the Quality Improvement Regulation.

### Theme III—Autonomy and adaptive capacity to tailor quality improvement efforts are imperative for impact

Governmental participants at the macro-level described the Quality Improvement Regulation as more relevant and suitable to various contexts compared to the previous Internal Control Regulations (see Table 7). Participants across all three system levels argued that the ability to improvise at a local level is key, as new situations continually take place [41, 49, 50]. Thus, having a regulatory solution of “one size fits all” is not recommendable. Micro-level findings in particular characterized autonomy as an enabler in any activity or quality enhancing effort in hospital management and practice—suggesting it impossible to anticipate and imagine every adverse event [49]. Autonomy could on one hand influence the enthusiasm of physicians to actively participate in systematic quality improvement work. On the other hand, autonomy could unfortunately leave the decision to implement incident reporting systems of the hospitals’ own choosing. Too many responsibilities and obligations left with hospital managers and a lack of time and resources to prioritize systematic PDSA methodology, missing competence, motivation, or simply disinterest, were reported as challenges to the regulatory implementation of quality and safety related requirements, and improvement efforts. The limited hospital manager training and support portrayed by inspectors and hospital managers at the meso, and micro-levels, added to these challenges. It moreover indicated that if managers were to find the regulatory framework instructive it was sometimes key to get help to “translate” legal terms.

Nevertheless, integrated findings across macro- and meso system levels displayed how professional, and administrative autonomy was viewed as imperative for the regulatory requirements to have any relevant impact on hospital practice and management. The synthesis also demonstrated that despite agreeing on autonomy as an important aspect to adaptive capacity in hospital implementation, the Quality Improvement Regulation was part of a wider regulatory context that excludes *full* autonomy. The latter relates to the principle of sound professional practice and prudent conduct, which in the Norwegian healthcare system is paramount to all aspects of the services offered. The integrated findings thus showed that hospital managers’ autonomy and adaptive capacity to tailor quality improvement efforts were dependent on other elements than requirements retrieved from the Quality Improvement Regulation alone.

## Discussion

Altogether this paper reports the synthesis of empirical data retrieved from the case study’s larger unit of analysis (the Quality Improvement Regulation), and its link to adaptive capacity across three system levels [10, 41, 49, 50]. The multilevel case study revealed how the Quality Improvement Regulation was developed, implemented, managed, and inspected across three system levels in the Norwegian healthcare context. Synthesized findings demonstrated that the Quality Improvement Regulation facilitated adaptive capacity, but nevertheless resulted in next to no change neither at the inspector level nor the hospital management level. This makes way for deliberation about how design, and implementation of healthcare regulation best links with adaptive capacity across system levels in order to result in relevant and efficient change and improvement. The latter relates to assumed conflicts between governmental expectations and managerial and supervisory implications [19, 28, 46, 56, 57]. The forthcoming discussion is divided into two parts, with links to all three themes.

### Implementing a context sensitive regulation followed up by a static inspection approach

Our study demonstrated that a regulatory regime supportive of contextual sensitivity does not guarantee actual implementation into inspectors’ nor managers’ work practices. We discuss these integrated findings by channeling: i) adapting regulation to ensure system resilience, ii) mismatch between regulatory changes and supervisory and hospital management practices, and iii) different scales of resilience.

#### Adapting regulation to ensure system resilience

The study overall identified that change was requested and needed, resulting in a regulatory revision. Synthesized findings indicated that the Ministry, and the Directorate, in documents and interviews, confirmed that embedded variation and complexity in healthcare settings required flexible regulatory expectations. This resonates with the resilience in healthcare concept in terms of acknowledging that external conditions may influence system performance, with real situations deviating from expectations [6, 19, 46, 47, 58, 59]. Thus, regulators in our study considered *work as done* when designing the Quality Improvement Regulation. A regulatory design that amongst regulatees is perceived to be sensible, could stimulate adaptive capacity across macro, meso and micro levels. Our synthesis finds that a collaboration between the system levels to mutually shape regulatory, supervisory, and management practices, may serve as an important collective potential to obtain system wide resilience. Past literature on the subject has highlighted this type of “mutual shaping” [60]. Furthermore, as a complex healthcare system is contained by uncertainty and variation, it seems key to ensure system wide adaptive capacity to meet with unexpected situations. The synthesis thus demonstrates a possible reconciliation between macro, meso, and micro-levels, by ways of seeing the various stakeholders in the system as collaborative partners in a regime of responsive regulation. This moreover implies that different stakeholders play mutual important roles in adapting regulation, to ensure the system’s resilience. This is a novel output in the research field because few studies have examined and contradicted the assumption that regulation and resilience are intractable opposites [6–9]. A regulatory and supervisory system that aims at regulating processes, through checking that hospitals are adequately undertaking quality improvement, can be considered both a better fit for a complex system and should also build resilience (and responsiveness to local conditions) through being more enabling of adaptations to local risks [6, 61]. As such, the performance-based regime investigated in our study, has great potential in being flexibly applied into hospital management and quality improvement processes. At the same time, it may build potential to foster a systematic process of hospital internal monitoring, anticipating, respond to, and learn from both regular work mode, and adverse events [45].

#### Mismatch between regulatory changes and supervisory and hospital management practices

The new Quality Improvement Regulation did not lead to changes in the inspectors’ work practices nor in the hospital managers’ work related to quality improvement activities. There was a mismatch between the introduction of the new Quality Improvement Regulation and the need to accordingly develop a new supervisory approach and provide hospital managers with sufficient support. The study hence indicated a potential to refine supervisory methods towards more regulator-regulatee interaction, increase the information exchange between inspectors and hospital managers and clinicians, put emphasis on developing methodology that helps hospital managers in “translating” supervision reports and frame problems into relevant improvement activities, as well as to increase attention to positive experiences and smart adaptations in hospital practice. This output vibrates implications in recent studies about innovation in supervisory methods in general, with special attention to involvement of next of kin and reflexive spaces [10, 15, 41, 49, 50, 62–66].

#### Different scales of resilience

Macro-level findings displaying the need for a regulatory change held together with micro-level findings, did show that several structural and cultural changes at the hospital level had occurred in recent years. We argue that these changes could link with resilience at different scales of organizational activity [67]. By applying these theoretical scales to our findings, macro-level findings indicated *systemic* resilience: demonstrating how the previous regulatory regime (the Internal Control Regulations) was reformed by the government into a new, PDSA designed Quality Improvement Regulation. This came as a response to needs proposed at the micro-level. Micro-level findings indicated *structural* changes as in for instance the recent establishment of different types of patient safety and quality councils in the hospitals. Our synthesis thus underpins that those processes to support change were partly ongoing regardless of the design and implementation of the new Quality Improvement Regulation and partly facilitated by performance-based regulation’s support for flexible management of quality and safety. We argue that responsive regulatory regimes foster adaptive capacity at different scales and therefore may contribute to promote collaboration among different stakeholder levels in healthcare [39, 40]. On the other hand, these integrated findings displayed a paradox as the systemic development was not followed by reported changes in management or supervisory practices.

### The complexity of autonomy in risk regulation and resilience

Our study demonstrated that autonomy and adaptive capacity to tailor quality improvement efforts were imperative if the regulatory framework ought to have any relevant impact on inspectors’ and managers’ activities. We discuss the integrated findings by channeling: i) the value of autonomy and management to successful implementation, ii) a regulatory craft focusing on responsive collaboration between regulators, inspectors and regulatees, and III) moving forward.

#### The value of autonomy and management to successful implementation

Performance-based regulation such as the Quality Improvement Regulation may represent an advantage compared to other regulatory strategies, in terms of supporting autonomy and adaptation to risk and context [10, 40]. As proved in our findings, hospital managers are expected to base their quality improvement measures and risk reducing activities on contextual conditions such as available resources and competences [30]. The ability to plan for, adapt to and anticipate local risks is thus incorporated into governmental expectations and regulatory strategies. As our study established a more nuanced outlook on regulation as governmental behavioral modification, the synthesis therefore counters with a traditional clinician viewpoint seeing regulation per se as a necessary evil and contradictory to autonomy and individual influence [6, 68]. On the other hand, autonomy and freedom to tailor improvement efforts require competence, additional resources, and systems to support hospitals and hospital managers, and responsible application by the regulatees [39, 40, 69, 70]. System’s “software”, including organizational culture, adequate management, and leadership, has shown to be a crucial determinant of quality improvement performance [61, 71]. For instance, micro-level noncompliance to implementation may link with a lack of management competences [72]. Attention to meaning and purpose in management training has therefore shown to be essential in achieving successful implementation in healthcare settings [20, 73–79]. As clinicians possess firsthand knowledge and experiences with adaptive capacity, past research has also demonstrated the urgency in having clinicians in management roles to get systematic improvement methodology embedded into everyday hospital work [77, 79–81].

Our synthesis, advocates for a more thorough understanding of how resources, competences and in-house interpretation support could constructively facilitate implementation of regulatory requirements and lead to long-term structural and systemic changes in the responsive healthcare system. We encourage further research to investigate into this field.

#### A regulatory craft focusing on responsive collaboration between regulators, inspectors and regulatees

By mapping the complex everyday reality in a regulatory regime in healthcare, our synthesis has sought out aspects that governments need to keep in mind while designing and developing new healthcare regulation.

Our study demonstrates that healthcare regulation is solely sensible if it is inclusive of those who are responsible to implement the strategies and requirements. Previous literature has shown how government audits with lack of attention to local conditions and stakeholder inclusion can lead to de-legitimization of external regulation [17, 40, 41, 79, 81–83]. In turn, lack of trust and unwillingness to learn can lead to all kinds of maladaptive behaviors [82]. As our micro-level findings displayed a lack of trust and motivation to learn from external inspection, it seems crucial that regulatory bodies play out a sensible “regulatory craft”. Their craft may influence the regulations’ impact on the regulatees’ behaviour and performance in terms of *how* regulatees implement a regulatory requirement or change [83]. Linked to our integrated findings, we uphold the argument that performance-based regulation like the Quality Improvement Regulation, seems sensible because it specifies preferences or objectives. It for instance recommends a PDSA approach, without compromising healthcare professional or institutional autonomy to choose appropriate actions and efforts according to relevant risks.

As different problems require different problem-solving techniques, the regulatory craft coincides with principles retrieved both from responsive regulation theory and resilience in healthcare [1, 2, 39, 40, 46, 83]. Central oversight, as in providing hospitals with a set of governmental requirements and regulations on one hand, and independent, local adaptations on the other, reflects how the two sets of ideas are entailed in any healthcare system. By ensuring adaptive capacity into the regulatory regime, it concurrently facilitates anticipation, another resilience potential [45]. Yet again, as our synthesis demonstrates, hospital managers’ ability to adapt and anticipate presuppose administrative and advisory support from the macro and meso-levels. Thus, these capacities’ purpose, namely, to corroborate system resilience, depend on central government support in budget allocations, as well as regional and local administered support to competence building and resources in general. Enabling factors that make performance management constructive have previously shown to be teamwork, self-organizing practices, and shared sensemaking [61]. These collaborative processes call for a double loop learning process. This is a core idea of the PDSA cycle, with focus on getting the information about organizational challenges as experienced by micro level stakeholders, further up the system chain, to meso and macro-level stakeholders [84, 85]. By having attention to information flow as part of the problem-solving, it may eventually lead to reevaluation of policies, regulations, and practices. In this, we find resonance with the resilience potential of learning, as well as connecting to the idea of trying to minimize the gap between work as regulators and inspectors often imagine it to be, and work as done by the regulatees [19, 46]. The PDSA cycle, as found in the design of the Quality Improvement Regulation, may contribute to stimulate reflection and accordingly, foster a recoupling between work as imagined and work as done. This reflexivity is important on all system levels (macro, meso, micro), as well as across system levels (macro-meso, meso-micro, macro–micro). Our synthesis therefore demonstrates that successful implementation presupposes a regulatory craft with focus on responsive collaboration, to reduce the mismatch found between regulatory changes and supervisory and hospital management practices. Performance-based healthcare regulation could consequently account for a balance between the ideal of centralization and the ideal of decentralization and contribute to adaptive capacity in hospital management of quality and safety. Thereby, the craft may foster system resilience.

#### Moving forward

Overall, what our synthesis showed was that to achieve a regulatory change, illustrated by the Quality Improvement Regulation, set out in a practical hospital context, adaptive capacity, is needed across three system levels. This includes more extensive collaborative efforts both prior to regulatory changes, during development and design processes, that exceeds the regular hearing processes. It also includes innovation to ensure collaboration between regulators and regulatees during external inspections, by increasing the application of expertise-oriented inspectors, recurring use of hospital self-assessment and involvement into implementation evaluation processes. Our study also illustrated that inspectors were expected to master the “craft” of moving between different regulatory strategies [10, 41]. The supervisory system examined may not have the sufficient construction to facilitate the responsiveness and trade-offs inspectors are supposed to handle. These aspects are important take home messages in the understanding of the thorough efforts required from stakeholders across all three system levels to improve quality and safety in healthcare settings. To ensure that the performance-based regulatory and supervisory model of which the Norwegian system builds upon, reflects the complexity it is supposed to regulate, inspect, and manage, we recommend a governmental evaluation of the implications our study has indicated. Our synthesis calls for a demanding systemic adaptive process, to move the current responsive regime forward.

### Strengths and limitations

The multilevel case study was based on the argument that various stakeholders at different system levels have different impact on the risk management process [47]. Through information flow and decision-making processes, the system levels are interwoven, implying that researchers need to understand the relationships among the different stakeholders [86]. One of the main strengths with a multilevel perspective is therefore the opportunity to investigate different realities and gain a systems perspective [5]. Accordingly, this synthesis paid attention to different realities at three system levels in the Norwegian healthcare setting. By assuming that integration could emerge from identification and tracking of macro, meso, and micro level participants’ views, expectations, and experiences of the *rationale* for the revised regulatory regime, in addition to actively look for links to *adaptive capacity* in the documentary evidence and interview responses, both bottom-up and a top-down perspectives were nurtured in this synthesis [87]. However, our study has some limitations. We did not limit our study to either specialized somatic healthcare or psychiatry but included managers from both areas. Considering differences in the arenas’ complexity, resources, and structures this could be considered a methodological limitation. The fact that the micro-level data collection included only two out of the four regional health authorities in the Norwegian specialized healthcare system, may have hampered our knowledge about geographical variations. Lastly, the discussion is based on the reality *as described by* the participants in the interviews, as well as evidence found in documents, not *as observed* by the researchers. Thus, our synthesis does not fully explain *why* changes in clinical and managerial behaviour was lacking.

## Conclusions

This paper represents a unique look into regulatory implementation across three system levels in healthcare, set out in a resilience perspective. In conclusion, and responding to the overall research question, integrated findings revealed that regulators at the macro-level and inspectors at the meso-level regarded variation, uncertainty, and complexity as key elements in hospital contexts, and thus important perspectives to consider during the design of the non-detailed Quality Improvement Regulation. The Quality Improvement Regulation was regarded to facilitate adaptive capacity. This contradicts the assumption that regulation per se and resilience are “hopeless opposites”. However, the core challenge with regulation is to provide healthcare professionals, clinicians, and their managers, with the relevant level of autonomy and competence to choose implementation efforts that are appropriate and relevant to the specific setting and context. Governmental expectations and inspectors’ methods were not fully recognized as efficiently and relevantly linked to hospital practice, management, and improvement methodologies, despite the new regulatory framework set out to support local quality and safety efforts in Norwegian hospitals. In that sense, the study identified a missing link in the current regime, related to differences in how inspectors and hospital managers viewed their collaboration and believed external inspection could improve quality and patient safety. We suggest that regulatory design and implementation processes, supervisory methods and hospital management practices could benefit from acknowledging adaptive capacity across macro, meso and micro levels as an important *collective* potential to obtain system wide resilience, and thereby ensure efficient risk regulation in quality improvement and patient safety processes. Responsive healthcare systems with care to a regulatory craft focusing on responsive collaboration between regulators, inspectors and regulatees, may have the prospect of overcoming the “sharp end” – “blunt end” dichotomy as an obstacle to implementation of quality improvement and safety enhancing activities. Further studies on regulatory design and development could explore how hospital management practices and implementation processes are influenced by the professional backgrounds and positions of regulators, inspectors, and managers, including their daily trade-offs to adapt to challenges and changes to maintain high quality care.

## Data Availability

Documents retrieved from online sources are publicly available. Documents exempted from public disclosure are not available. Data retrieved from the interviews is available from the corresponding author upon reasonable request and with permission from the participant(s).

## Abbreviations

Internal Control Regulation: Internal Control Regulations in the Healthcare Services
PDSA: Plan, do, study, act
The Ministry: The Ministry of Health and Care Services
The Inspectorate: The Norwegian Board of Health Supervision
The Directorate: The Norwegian Directorate of Health
The Quality Improvement Regulation: Regulation on management and quality improvement in the healthcare services
